# Carbon nanoparticles localized clipped node dissection combined with sentinel lymph node biopsy with indocyanine green and methylene blue after neoadjuvant therapy in node positive breast cancer in China: initial results of a prospective study

**DOI:** 10.1186/s12957-023-03120-8

**Published:** 2023-07-21

**Authors:** Xin Yang, Yao Li, Xiao-tian Ren, Lei Fan, Bin Hua

**Affiliations:** Breast Center, Department of Thyroid–Breast–Hernia Surgery, Department of General Surgery, Beijing Hospital, National Center of Gerontology, Institute of Geriatric Medicine, Chinese Academy of Medical Sciences, No. 1 Dahua Road, Dongcheng District, Beijing, 100730 People’s Republic of China

**Keywords:** Targeted axillary dissection, Sentinel lymph node biopsy, Neoadjuvant therapy

## Abstract

**Background:**

This study aimed to evaluate the feasibility of applying carbon nanoparticles (CNs) to visualize clip-marked metastatic nodes in combination with indocyanine green (ICG) and methylene blue (MB) as sentinel lymph node (SLN) tracers for targeted axillary dissection (TAD) after neoadjuvant therapy (NAT).

**Methods:**

This feasibility trial enrolled 40 patients with node-positive breast cancer, and 38 patients completed NAT and surgery were included in the final analysis. Before NAT or surgery, clip-marked nodes were localized with CNs by ultrasonography. After NAT, the clip-marked nodes were removed under the guidance of carbon-tattooing and confirmed by radiography. SLNs were mapped with ICG and MB. Axillary lymph node dissection (ALND) was performed for all patients after TAD.

**Results:**

The clip-marked nodes were retrieved in all patients. The SLN identification rate was 100%. 29 of 38 clipped-nodes were SLNs. The false-negative rate was 6.25% (2/32,95% CI:1.09% ~ 22.22%) for TAD nodes and 9.38% (3/32,95% CI 3.24%-24.22%) for SLNs, and 18.75% for clipped-nodes (6/32, 95% CI:7.86% ~ 37.04%). No adverse events were reported during clip placement, CNs localization, or the TAD procedure.

**Conclusions:**

The TAD procedure with CNs to locate and visualize clipped nodes as well as SLN tracing with ICG and MB has good accessibility in China, and this technique could be easily mastered by experienced surgeons. The modified TAD technique has also demonstrated good predictive ability for residual axillary lesions after NAT, and is worth of further evaluation.

## Introduction

Neoadjuvant therapy (NAT)is the standard protocol for breast cancer patients [1–3]. It has been reported a targeted axillary dissection (TAD) procedure, a comprehensive method to stage axilla after NAT by dissecting clipped-metastatic nodes before NAT in addition to performing SLNB during surgery which could improve the accuracy of axilla evaluation after NAT [4–7]. Beyond wires and ^125^I seeds, the SAVI SCOUT® reflector and magnetic seeds have also been proven to be effective in localizing metastatic nodes for TAD during surgery [8, 9]. However, the reported TAD procedures were difficult to implement in China. First, the locating wire is easily dislocated and may not be well tolerated by patients. Second, ^125^I seeds and radioisotope tracer require special licenses for safe use. Third, the SAVI SCOUT® reflector and magnetic seeds are unavailable in China.

Carbon nanoparticles (CNs) are a less expensive localizer for metastatic nodes that can identify 75% of targeted nodes in a median of 183 days after NAT when used alone [10], and are widely used in China for lymph node tracing in various tumors [11–13]. And no additional localization is required for the tattooing technique of positive node before and during operation. SLN mapping with indocyanine green (ICG) has become popular in China because of its fast nature, and comparable accuracy with traditional tracers when used in breast cancer patients with upfront surgery [14]. However, as a part of TAD, whether ICG based SLN mapping is accurate after NAT has not been verified. All of these issues suggest that we need to modify the TAD procedure in China.

We hypothesized that CNs could locate and visualize clipped-nodes during surgery, and ICG with MB could map SLNs effectively. The goal of this prospective pilot study was to explore the feasibility of this modified TAD procedure in China.

## Materials and methods

### Ethics and patients

This prospective single-center feasibility study was approved by the Ethics Committee of Beijing Hospital (IRB Number for ethical approval: 2020BJYYEC-062–03), and was registered in the Chinese Clinical Trial Registry on June 3, 2020. (http://www.chictr.org.cn identifier: ChiCTR2000033505)**.** Forty patients with biopsy-proven node metastases were enrolled in the study. Clinical and pathologic data were collected from medical records. Patients with distant metastases, ipsilateral supraclavicular/infraclavicular and internal mammary lymph node metastasis or prior axillary surgery, including SLNB, were excluded.

### Clip placement and CNs localization with ultrasound

**Fig. 1:**
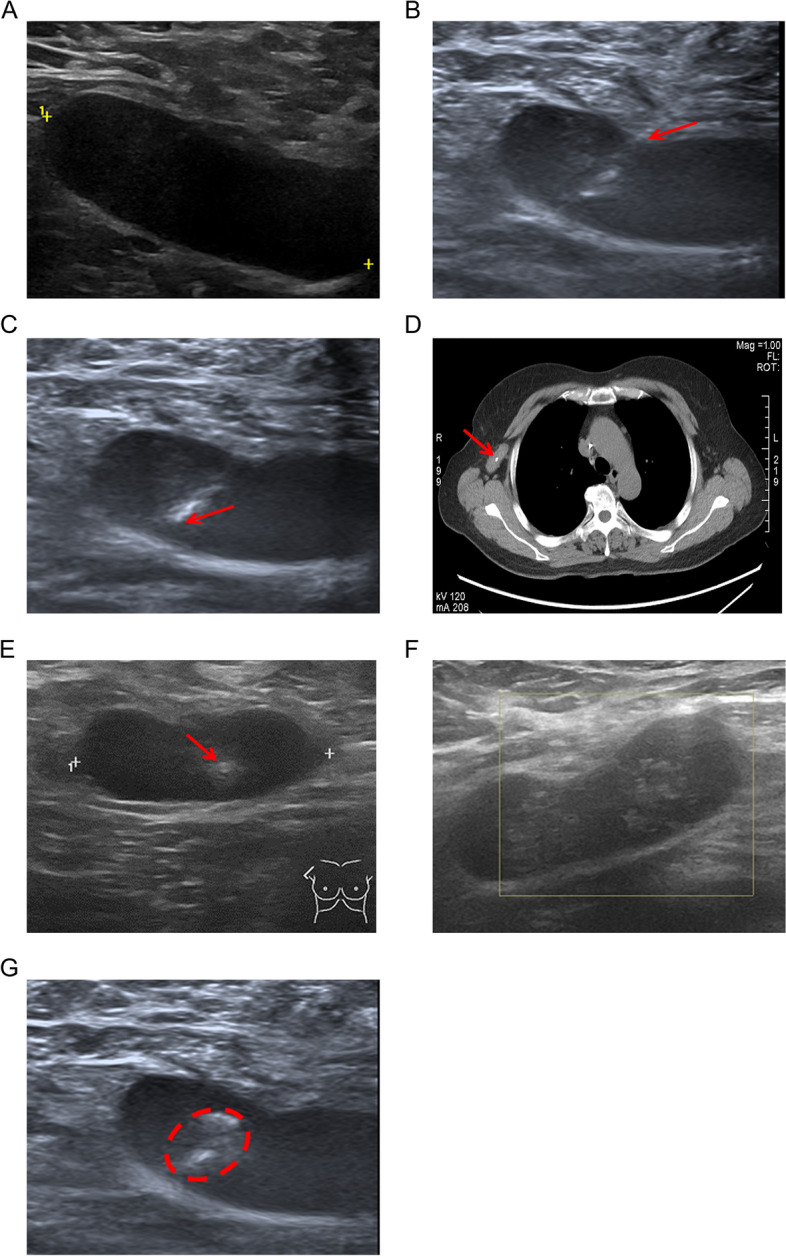
The procedure for marking the biopsy-proven metastatic lymph nodes with clips and carbon nanoparticles (CNs) guided by ultrasound. **A** An abnormal lymph node was found by ultrasonography and confirmed by fine-needle biopsy. **B** The process of placing a Bard clip propeller in the biopsy-proven node, and the arrow shows the puncture route. **C** The hyperechoic clip (arrow) in the hypoechoic lymph node. **D** The clip (arrow) inside the axillary lymph node detected by preoperative chest computed tomography. **E** After 2 courses of neoadjuvant therapy, the clip (arrow) in the lymph node can be seen by ultrasonography. **F** Because of fibrosis after neoadjuvant therapy, it was difficult to distinguish the clip by preoperative ultrasound after 6 courses of neoadjuvant therapy. **G** After the injection of 0.05 ml CNs, a hyperechoic region (circle) appeared around the clip

Bilateral regional lymph node basins, including the axilla, infraclavicular and supraclavicular node basins, were checked by ultrasonography in patients with breast cancer. When enlarged internal mammary lymph nodes were found by chest computed tomography or breast magnetic resonance imaging, ultrasonography was performed for confirmation. When abnormal lymph nodes [15] were found, patients underwent fine-needle aspiration biopsy with a 25-gauge needle. After metastatic disease was confirmed in the node, a clip (ULTRACLIP, Bard Peripheral Vascular Inc., Arizona, USA) was placed in the lymph node cortex (Fig. 1A, B and C).

In the initial six patients, 0.05–0.5 ml CNs (Chongqing Lummy Pharmaceutical Co., Ltd., Chongqing, China) were injected around the clip the day before operation. Although pre-operative chest computed tomography could easily detect a clip in the lymph node (Fig. 1D), it was challenging to localize the clipped-nodes after NAT by ultrasound (Fig. 1E and F). Because carbon dye can be retained in tissue for a long time [10], from the seventh patient, the CNs were injected around the clip immediately after clip labeling (Fig. 1G). In addition, 0.05 ml of CNs was set as the final injection dose. More volume would lead to overflow of the CNs from the lymph nodes, resulting in extensive black staining of the axillary contents.

### Treatment

Patients received neoadjuvant chemotherapy (anthracycline- and taxane-based regimens) or combined with targeted therapy (trastuzumab and pertuzumab regimen) based on the molecular subtypes. After NAT, TAD procedure, breast surgery and Axillary lymph node dissection (ALND) were performed during the same session.

#### SLNB procedure

**Fig. 2:**
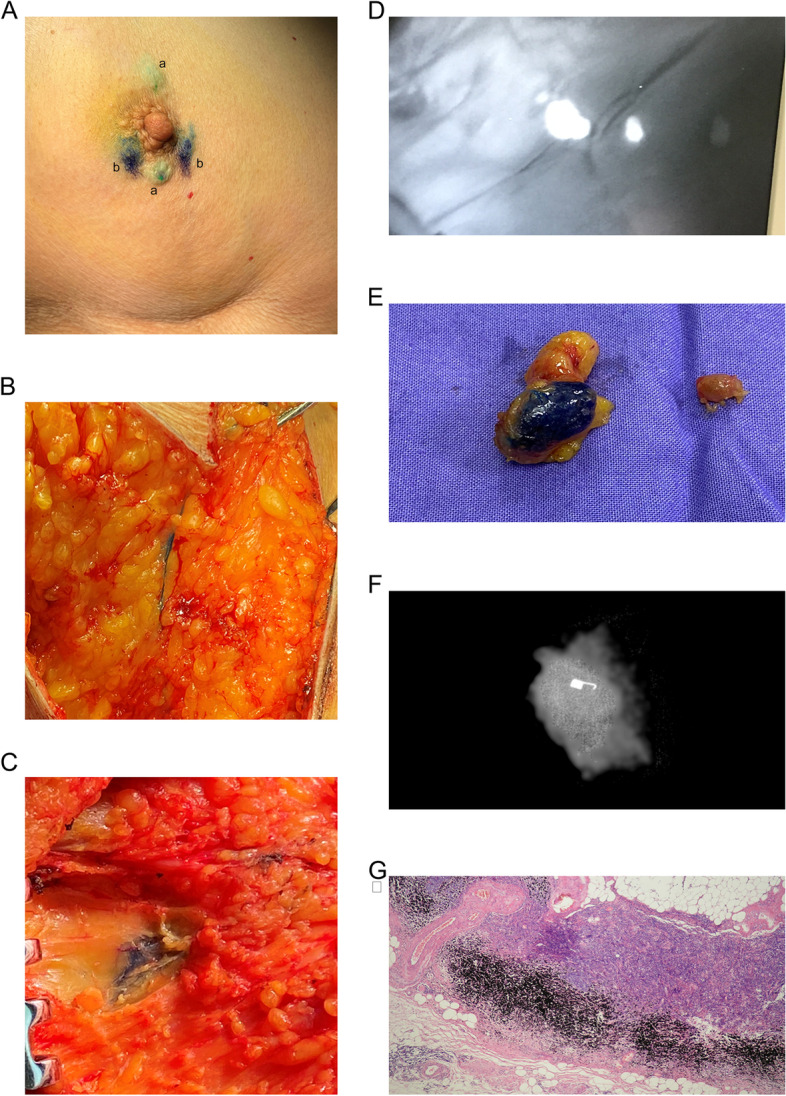
The modified targeted axillary dissection procedure. **A** Intradermal injection of indocyanine green (ICG) (a) and methylene blue (MB) (b) around the areola before the operation. **B** Lymphatic vessels stained mainly with MB. **C** The adipose tissue stained by carbon nanoparticles (CNs) overflowing in the axillary tissue, with the clipped lymph node below.** D**, **E** ICG traced two sentinel lymph nodes (SLN), and CNs and MB stained one SLN. **F** Clip in the CNs-located node confirmed by intraoperative mammography. **G** Carbon pigments in the lymph node subcapsular sinus (hematoxylin–eosin × 40)

0.5 ml MB at a final concentration of 2% (Jumpcan Pharmaceutical Group Co., Ltd., Taixing, China) and 1 ml (0.75 mg) ICG (Dandong YICHUANG Pharmaceutical Co., Ltd., Liaoning, China) were used for SLN mapping as described previously (Fig. 2A) [16].

#### Surgical procedure and pathologic evaluation

During the operation, the axillary lymph node basin was explored, and the clipped-node was located and removed according to tattooing and confirmed by intraoperative radiography, and fluorescent, blue-stained, or palpably abnormal nodes were considered SLNs (Fig. 2B-F). The relationship between the clipped node and SLNs was determined according to the presence of fluorescence and/or blue staining in the clipped node. The mapping patterns of each SLN were recorded in detail. All patients underwent ALND after TAD.

A separate pathologic diagnosis of the clipped-node (Fig. 2G) and SLNs was given in the pathologic report. Any residual disease, was considered to indicate a positive lymph node.

### Statistical analysis

IBM-SPSS version 22.0 (IBM Corp., Armonk, NY, USA) was used for data collection and analysis. False negative rate (FNR) of targeted nodes, SLNs and clipped nodes was preliminarily analyzed. FNR was equal to the number of patients with false-negative metastatic nodes divided by the total number of patients with residual node disease.

## Results

### Clinicopathological data of the enrolled patients

From July 2020 to March 2022, forty patients with node-positive disease were eligible for the trial. As of July 2022, thirty-eight of the patients had completed NAT and surgery, one patient did not undergo operation, and the other one withdrew informed consent. The clinicopathologic data of the thirty-eight patients were shown in Table 1.

**Table 1:** Clinicopathological characters of the enrolled patients (*N* = 38)

Parameters	N (%)
Age (median (range))	53 (36–69)
Grade	
II	28 (73.68)
III	10 (26.32)
Subtypes	
Luminal A	6 (15.79)
Luminal B HER-2 negative	12 (31.58)
Luminal B HER-2 positive	7 (18.42)
HER-2 positive	9 (23.68)
TNBC	4 (10.53)
Anatomical stage before neoadjuvant therapy	
IIA	6 (15.79)
IIB	24 (63.16)
IIIA	8 (21.05)
Tumor histopathology	
Ductal	35 (92.11)
lobular	1 (2.63)
Other type	2 (5.26)
Neoadjuvant systemic regimen	
Chemotherapy	24 (63.16)
Chemotherapy and targeted therapy	14 (36.84)
Surgery	
Breast-conserving therapy	3 (7.89)
MRM	31 (81.58)
MRM + Expander implantation	4 (10.53)
Interval from clip + carbon nanoparticles injection to operation (day), median (range)	127 (1–203)
Clinical response evaluation based on RECIST	
Complete response	5 (13.16)
Partial response	26 (68.42)
Stable disease	7 (18.42)
Progression of disease	0 (0)
Miller-Payne grade system	
G1-G2	18 (47.37)
G3	9 (23.68)
G4	6 (15.79)
G5	5 (13.16)
Residual cancer burden index	
0	3 (7.89)
I	4 (10.53)
II	12 (31.58)
III	19 (50.00)
Clipped node in SLNs	29 (76.32)
Distribution of residual node disease(*N* = 32)	
TAD nodes only	14 (43.75)
TAD nodes + other axillary nodes	16 (50.00)
Other axillary nodes only	2 (6.25)

*Abbreviations*: *HER-2* Human epidermal growth factor receptor 2, *MRM* Modified radical mastectomy, *SLN* Sentinel lymph node, *TNBC* Triple negative breast cancer, *RECIST* Response evaluation criteria in solid tumors, *TAD* Targeted axillary dissection

### The modified TAD technique was feasible after NAT

The median interval between CNs injection and surgery was 127 days (range 1–203 days). All patients had successful CNs visualization and surgical removal of the clipped nodes, as confirmed by intraoperative radiography. Three surgeons in our center participated in the study, and these surgeons completed 5, 10, or 23 operations respectively. All of them successfully found the clipped metastatic lymph nodes according to the presence of carbon tattooing. The dual tracing technique with ICG and MB successfully located the SLNs in all patients.

### The modified TAD technique had the potential for staging axilla after NAT accurately

**Fig. 3:**
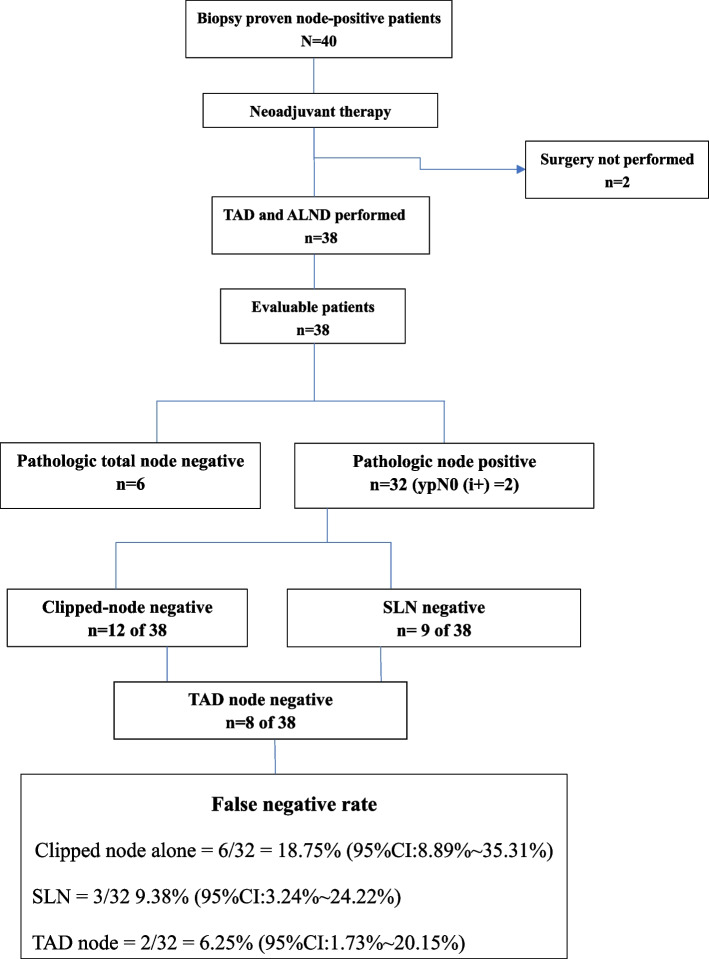
Ability of clipped nodes, sentinel lymph nodes (SLNs) and nodes removed by targeted axillary dissection (TAD nodes) to predict axillary nodal status after neoadjuvant therapy

The median number of SLNs and TAD nodes was 5 (SLN: range 2–6; TAD: range 2–8), and the clipped nodes were SLNs in 29 patients. The final pathologic evaluation determined that 6 patients achieved pathological complete response (pCR) in axilla (15.79%,6/38) and 32 patients had residual nodal disease (84.21%), of whom one patient had isolated tumor cells (ITCs) in one of TAD nodes and another patient had ITCs in two of the remaining axillary lymph nodes. 12 patients achieved pCR in clipped nodes, 9 patients achieved pCR in SLNs and 8 in TAD nodes. The FNR was 18.75% (95%CI:8.89% ~ 35.31%) for clipped nodes (6/32), 9.38% (95%CI 3.24% ~ 24.22%) for SLNs (3/32), and 6.25% (95%CI:1.73% ~ 20.15%) for TAD nodes (2/32) (Fig. 3; Table 1). The modified TAD technique demonstrated the ability to accurately evaluate the status of axillary lymph nodes after NAT with a high identification rate and a low FNR of TAD nodes.

**Fig. 4:**
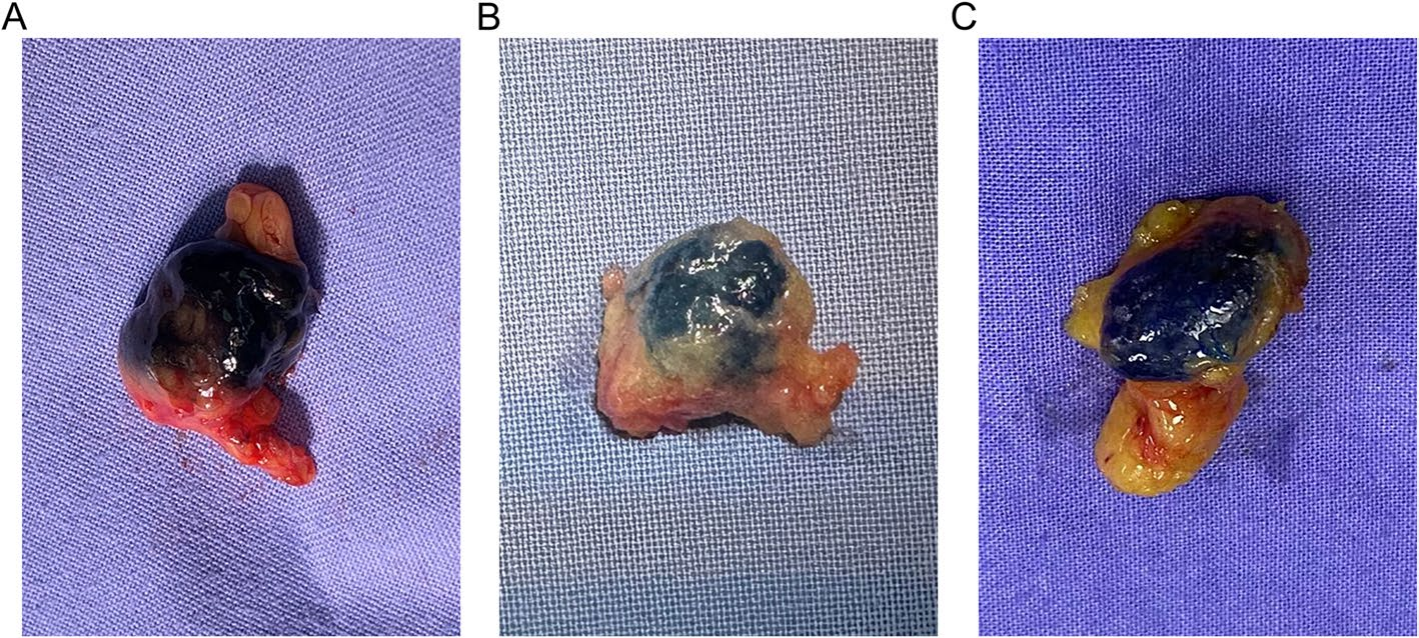
Carbon nanoparticles (CNs) might interfere with the interpretation of methylene blue (MB) tracing effect due to color similarity. **A** Lymph node stained by CNs alone. **B** Lymph node stained by MB alone. **C** Lymph node stained by CNs and MB simultaneously

No adverse events were reported during clip placement, CNs localization, or the TAD procedure. When clip-labeled lymph nodes were SLNs, it’s necessary to distinguish between the carbon black and navy blue of MB carefully (Fig. 4A and B), but it would not affect mapping of the SLNs by ICG (Fig. 2D) or staining of the lymphatic vessels (Fig. 2B).

## Discussion

The TAD procedure has been proven to be an effective method to stage axilla after NAT in breast cancer patients with axillary metastatic disease and provides strong support for individualized axillary treatment (Table 2) [17–20]. The intraoperative localization method of the clipped nodes before NAT and SLN tracing are the key points of TAD. The previously reported methods are difficult to carry out in China due to material accessibility or technical problems [7, 8, 18]. Therefore, we need a modified TAD procedure with good accessibility in China to provide precise axillary staging after NAT. We designed this prospective study with CNs to mark the clipped nodes before NAT and visualized the labelled nodes with carbon black, and with ICG and MB to map the SLN in operation. This is a preliminary result to analyze the feasibility of this modified TAD technique in China, and evaluations of the accuracy of this method in predicting residual axillary lymph node disease after NAT is under way.

**Table 2:** Studies that have analyzed the feasibility of marked lymph nodes and /or SLN to stage axilla after NAT

Author	Year	No. of patients^a^	SLN IR (%)	Tracers for SLNs	Marked node and/or SLN IR	Matched rate of marked nodes with SLNs (%)	Methods to visualize marked nodes	FNR (%)	Median of SLN
Donker, et al [13]	2015	100	-	-	97	-	^125^I seed	7 for MARI nodes	-
Caudle, et al [14]	2016	85	95.5	BD + RI OR RI	94.8	77	^125^I seed	2 for TAD nodes 4.2 for clipped nodes 10.1 for SLN	2.7
Cabioglu, et al [15]	2018	98	87.8	BD + RI (*N* = 37) BD (*N* = 61)	95.6	81.4	Specimen radiographs	11.4 for all 4.7 for clipped + doubel tracing	2
Simons, et al [20]	2022	212	86.4	BD + RI	98.2	71.3	^125^I seed	3.5 for RISAS nodes 17.6 for SLN 7 for MARI nodes	/
This article	2023	38	100	ICG + BD	100	76.3	carbon nanoparticles	6.25 for TAD nodes 9.38 for SLN 18.75 for clipped nodes	5

*Abbreviations*: *FNR* False-negative rate, ^*125*^*I* Iodine-125, *IR* Identification rate, *MARI* Marking the axillary lymph node with radioactive iodine seeds, *NAT* Neoadjuvant therapy, *RISAS* Radioactive iodine seed localization in the axilla combined with sentinel node procedure, *SLN* Sentinel lymph node, *TAD* Targeted axillary dissection

^a^This includes all patients in whom ALND is performed with TAD

CNs are widely used in China for lymph node tracing of various tumors with reasonable prices [11–13]. In this study, we used CNs labeling to realize the intraoperative visualization of clipped nodes, and no additional equipment or location is required during or before operation. Under the guidance of carbon tattooing, all clipped nodes were found during the operation, improving the identification rate of the biopsy-proven nodes over that achieved with carbon dye alone [10, 21]. In addition, CNs injections can be performed before surgery or before NAT, but it is easier to locate the metastatic lymph nodes if the injection occurs before NAT, and consistent with the literature, CNs can maintain the tattooing efficiency after a median of 127 days (range 1–203 days) [10, 21].

There is no consensus on the volume of carbon dye that should be injected in different studies. Researchers believe that different doses can be selected based on lymph node size and individual experience [10, 21, 22]. But too much carbon dye can lead to extensive staining of the axillary tissue and CNs migration to other nodes [10]. When 0.5 ml of CNs was used to mark the clipped node in the first two patients, we found the nodes in level II blacked in one patient. No CNs migration was found when the injection volume of CNs decreased below 0.5 ml. Through the clinical observation of the first six patients (0.5 ml for two patient, 0.1 ml for two patient, and 0.05 ml for two patients), we found that 0.05 ml of CNs could locate the clipped nodes, had little effect on the axillary tissue from injection overflow, and had no CNs migration. Finally, we determined 0.05 ml CNs as our final working volume, which successfully labeled the clipped nodes in the subsequent 32 patients without affecting the SLN tracing results.

CNs did not affect fluorescent tracing. Similar to the previous literature [21], the color similarity between the CNs and MB made it difficult to distinguish lymph nodes stained with both at the same time, but there was no effect on the MB staining of other SLNs or lymphatic vessels if no excessive overflow of CNs. In addition, there are few patients with tattoos in China, so there is a low likelihood that localization of the clipped nodes with CNs would be affected by other factors.

It has been reported that the accuracy of SLNB by radioisotope and/or blue dye (BD) decreased after NAT, which manifests as a decrease in the detection rate and an increase in the FNR [1, 4, 5]. Chirappapha et al. found that the accuracy of SLN tracing with ICG and BD is superior to that with radioisotopes and BD after NAT [23]. In this modified TAD, we used ICG and MB to map SLN after NAT. Both identification rates of TAD nodes and SLNs were 100%. only two patients with negative TAD nodes had residual disease in the rest nodes of axilla, and the FNR was 6.25%. In addition, only three patients with negative SLNs had metastatic disease in the rest axillary lymph nodes, and the FNR was 9.37% (3/32). These results suggested that the modified TAD technique had the potential for staging axilla after NAT accurately, and SLNB guided by ICG and MB had good accuracy in axillary evaluations, and whether ICG + MB dual tracing could simplify the individualized axillary evaluation should be further explored in patients with node-positive breast cancer after NAT.

This study has some limitations. First, the sample size was too small to evaluate the power of each axillary staging procedure. Second, the color confusion between CNs and MB was objective, and large sample Cohort study is needed to analyze the role of MB in this modified TAD technique.

## Conclusions

Locating the biopsy-proven clipped lymph nodes with CNs before or after NAT in combination with SLNB by ICG and MB is feasible to evaluate armpits after NAT. The materials and tracers involved in our study are accessible in China, do not need special sites or staffing, and have good prospects for surgical application. A profound study with a large population in our center is underway to determine the clinical implications of this modified TAD including long-term side effects and its oncological safety.

## Data Availability

All data generated or analyzed during this study are included in this published article.

## Abbreviations

CNs: Carbon nanoparticles
ICG: Indocyanine green
MB: Methylene blue
SLN: Sentinel lymph node
SLNB: Sentinel lymph node biopsy
TAD: Targeted axillary dissection
NAT: Neoadjuvant therapy
ALND: Axillary lymph node dissection
FNR: False-negative rate
pCR: Pathological complete response
ITCs: Isolated tumor cells
BD: Blue dye
HER-2: Human epidermal growth factor receptor 2
MRM: Modified radical mastectomy
TNBC: Triple negative breast cancer
RECIST: Response evaluation criteria in solid tumors
